# Fast and Secure Multiparty Querying over Federated Graph Databases

**DOI:** 10.1007/s42979-025-04503-2

**Published:** 2025-11-28

**Authors:** Nouf Aljuaid, Alexei Lisitsa, Sven Schewe

**Affiliations:** 1 Department of Information Technology, College of Computers and Information Technology, Taif University, P.O. Box 11099, Taif, 21944 Saudi Arabia; 2 Department of Computer Science, University of Liverpool, Liverpool, UK

**Keywords:** Neo4j, Graph databases, Secure multi-party querying, Federated databases, SMPC

## Abstract

We have developed a framework for efficient privacy preserving multi-party querying (PPMQ) over federated graph databases, leveraging Secure Multi-Party Computation (SMPC) protocols to enhance data security. The system offers two distinct security protocols: a client-based protocol and a server-based protocol. In the client-based protocol, standard SMPC techniques are employed, allowing computations to be performed on data without exposing the data itself. The server-based protocol employs SMPC to facilitate secure data processing and is further enhanced by encrypted hashing, which adds an additional layer of security to prevent data exposure. We conducted experiments comparing PPMQ with Neo4j Fabric and two previous systems, SMPQ and Conclave. The results indicate that PPMQ’s execution times and overheads are comparable to those of Neo4j Fabric, while outperforming both SMPQ and Conclave, demonstrating its superior efficiency. Additionally, PPMQ, like SMPQ and Conclave, utilises an honest but curious security model. However, it enhances the security of the server protocol, making it more robust against brute force attacks and providing stronger privacy guarantees than previous solutions.

## Introduction

Data security is a crucial aspect and one of the fundamental challenges faced by IT professionals. Over the years, numerous techniques have been proposed and implemented to enhance data security [33]. One such example is secure multi-party computation (SMPC), which, according to [13], is a cryptographic technique that allows a group of individuals to perform computations collectively without revealing their individual data to one another. The primary benefit of using such a technique is that it enables cooperation and coordination in environments where such collaboration was previously impossible due to trust issues [23]. This method offers several advantages, including ensuring that no third parties, even those considered trustworthy, can access the data. It effectively eliminates the need to choose between data privacy and usability, enabling accurate processing without compromising confidentiality.

Nowadays, SMPC is employed in a wide variety of practical contexts, including the detection of financial fraud [41], the aggregation of model features across private datasets, and the prognosis of heart disease [46]. Additionally, it can assist in resolving trust issues in situations such as secure elections [6], auctions [7], and secret sharing [16].

Another application of SMPC is to secure the query on different types of databases. Up until now, SMPC has primarily been utilized for safeguarding relational databases [8, 10, 48]. Although SMPC is effective in securely executing queries, its application in the context of graph databases has been minimally explored. The authors of [12] employs SMPC at the backend, while queries remain one-party only and secure multi-party querying (SMPQ) [1] which implements multi-party queries.

In comparison to relational databases, graph databases offer a more flexible data model that proves more efficient for certain types of queries, such as traversal queries [40]. The graph-based structure utilised by these databases has demonstrated advantages in various contexts and has been widely adopted in platforms like Instagram, Twitter, and Facebook [12].

Although SMPC applied to multi-party queries holds potential, significant challenges persist, particularly in terms of performance. For instance, our SMPQ system, discussed in [1], builds on the previously developed Conclave system [48] for graph databases and improves processing, yet considerable overheads remain.

In this work, we present a privacy-preserving multi-party query system (PPMQ) designed to enhance security and efficiency in querying federated graph databases. PPMQ leverages the Neo4j Fabric functionality, extended with the Awesome Procedures on Cypher (APOC) library, to facilitate complex queries while maintaining data confidentiality. A primary motivation for focusing on graph databases is their inherent strength in modeling and querying complex relationships, which are common in many federated data scenarios. While the initial validation of PPMQ in this work employs foundational query types to establish the core privacy-preserving mechanisms, the choice of a graph database architecture, specifically Neo4j, is strategic for our long-term vision of supporting complex graph-native analyses (such as traversal queries) in a federated, privacy-preserving manner.

This system offers a customisable and adaptable approach to privacy preservation through two distinct security protocols: a client-based protocol employing traditional SMPC techniques, and a server-based protocol that integrates SMPC with encrypted hashing to safeguard against data exposure.

This paper extends the work presented in [4] by introducing several key improvements. It offers a more comprehensive overview of existing SMPC implementations, including detailed explanations of the secret-sharing method used in JIFF. Furthermore, we have expanded the system overview to include requirements and the threat model. The server protocol has been enhanced to use HMAC hashing with a derived key for improved security, and a Preliminaries section has been added to explain the operations used in the protocols. Finally, the System Evaluation section has been extended to study scalability and has been updated with revised experiments and results.

The remainder of this paper is organised as follows: the following Sect. 2 presents the background to this work, followed by related existing works in Sect. 3. The design of the proposed approach is detailed in Sect. 4. Next, Sect. 5 explains how the system works. Following this, Sect. 6 outlines the query flow within the proposed system, with the system evaluation and results of our experiments presented in Sect. 7. Finally, the paper concludes in Sect. 8, where we also provide directions for future work.

## Background

### Secure Multi-Party Computation (SMPC)

SMPC is a cryptographic method that distributes computing tasks among multiple parties to prevent any individual from accessing or figuring out confidential information belonging to other participants, as stated in [13]. It enables a group of participants to collaborate and perform a computation on their data that they all agree upon while preserving the confidentiality of their respective inputs.

**Fig. 1:**
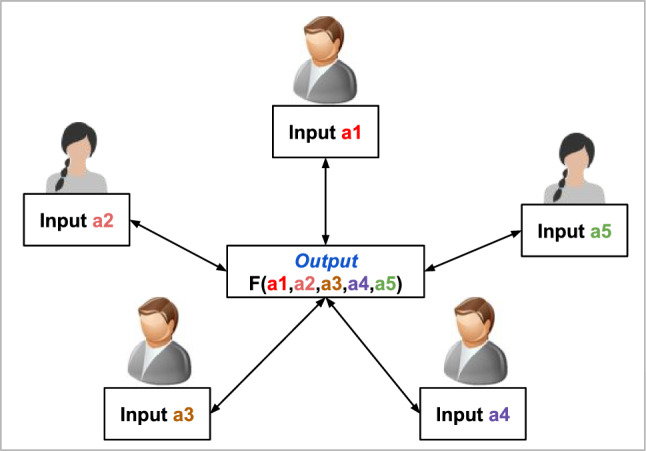
Multi-party computation among five parties

In a scenario where a set of parties, denoted as $\textsf{P}$, each having confidential data $\textsf{X}$, aim to collaboratively calculate a function $\textsf{F}$ based on their combined inputs, a challenge arises because they do not trust one another and seek to safeguard their inputs. If the parties could identify a trustworthy third party, $\textsf{Z}$, they would need to disclose their data to $\textsf{Z}$, who would then execute the function on their behalf and share the outcome, $\textsf{Y}$, with them. However, an SMPC protocol provides a solution to compute $\textsf{F}$ securely, ensuring that the output matches what $\textsf{Z}$ would have obtained. Figure 1 illustrates a generic scheme of SMPC among five parties.

### Neo4j and Cypher Query Language

In this work, we have used Neo4j [20], one of the most widely used implementations of graph databases. It employs a data model known as labeled property graphs, which consist of nodes and relationships. Nodes represent individual entities, while relationships define directed, meaningful connections between nodes, typically illustrated with arrows [31]. Each node can have properties, which are key-value pairs that store additional information about the entities. Additionally, nodes can be assigned labels that indicate their roles, making searches within the graph simpler and more efficient.

Neo4j uses the Cypher query language to interact with graph-structured data [17]. To further enhance its capabilities for federated data scenarios, Neo4j Fabric [19] allows the execution of Cypher queries across multiple Neo4j graph databases simultaneously, making it well-suited for federated database environments. Fabric operates by creating a virtual layer known as a fabric database, which coordinates queries over the underlying databases. This setup enables users to access and analyse data as if it were within a single, unified graph, even when the data is distributed across multiple databases [37].This intrinsic capability of Neo4j Fabric to query across distributed graph datasets seamlessly is a key factor in selecting Neo4j for PPMQ, directly aligning with the architectural requirements of a federated system designed for graph-structured data. It provides a robust foundation that is not readily available in traditional relational database systems when considering federated graph-specific operations. Additionally, the APOC library [35] provides over 450 procedures and functions that support various tasks such as data integration, transformation, graph algorithms, and other operations not natively supported by Cypher.

**Fig. 2:**
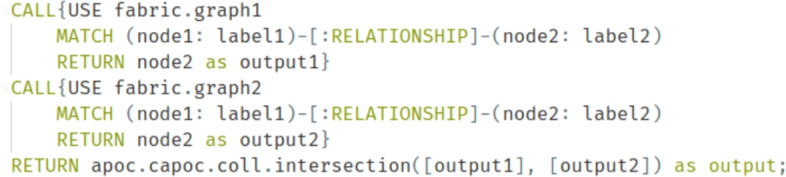
Example of a general cypher query utilising two parties with two databases [4]

Figure 2 illustrates an example of a general Cypher query that demonstrates a federated query across two graph databases using Neo4j Fabric and the intersection from the APOC library.

As shown in the example, the structure of the query involves two calls to different graphs, enabling querying across multiple databases managed under the same Neo4j Fabric instance. Subsequently, the intersection of the results from each database is applied, allowing for the identification of common nodes.

## Related Work

### Implementation of SMPC

Until recently, the main emphasis of research on SMPC has been theoretical. However, there has been a substantial effort to apply SMPC in practical real-world scenarios. [16]. Various implementations of SMPC, including JIFF [3], Oblivm [29], GraphSC [34], Sharemind [11], ABY [15], MP-SPDZ [26], EMP-toolkit [49], TinyGarble2 [25], and Frigate [32], have been utilised in different contexts.

**JIFF** [3] is a JavaScript library designed for scenarios where data is distributed among multiple entities, enabling SMPC. It operates under an honest-but-curious security model, using a server to manage the exchange of encrypted messages between participants. JIFF employs Shamir’s secret sharing scheme [42] to facilitate secure computations by dividing each participant’s private data into shares that are distributed across multiple parties. Individually, these shares are meaningless but can be combined to reconstruct the original secret.

**Fig. 3:**
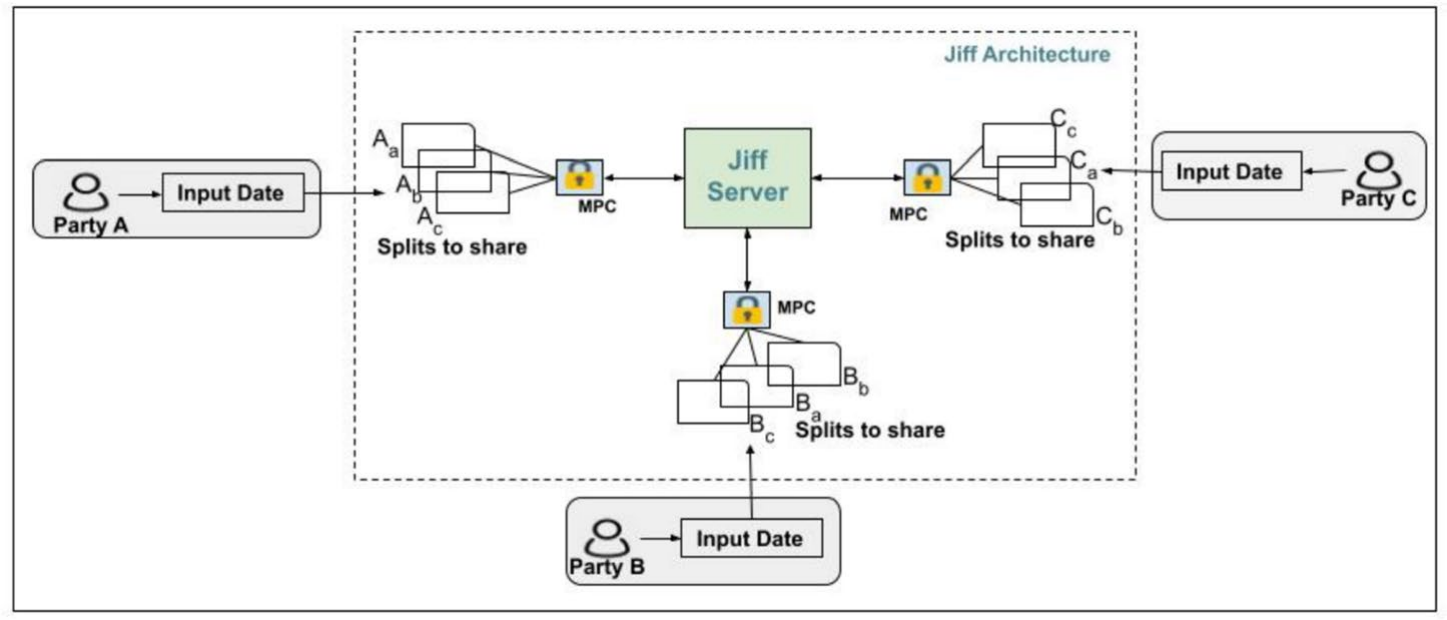
JIFF architecture and components [4]

During the computation process, the input data from participants is split into shares, which are then encrypted using the public keys of the involved parties. Each participant holds shares from other parties in addition to their own. Computations are performed on these encrypted shares, and the results are displayed to all participants, ensuring that the underlying data remains confidential throughout the process. The architecture of JIFF is depicted in Fig. 3.

#### Shamir’s secret sharing

The protocol involves two main operations: share generation and reconstruction [42]. During share generation, shares are created from a given secret input using a polynomial equation, with the secret embedded as the constant term of the polynomial. The polynomial is evaluated at distinct points to produce the shares, which are then distributed among *n* parties. The reconstruction process involves collecting a minimum number of shares, defined by the threshold *k*, to accurately reconstruct the original secret input using polynomial interpolation. The details of both operations are presented at Algorithm 1.

**Algorithm 1:**
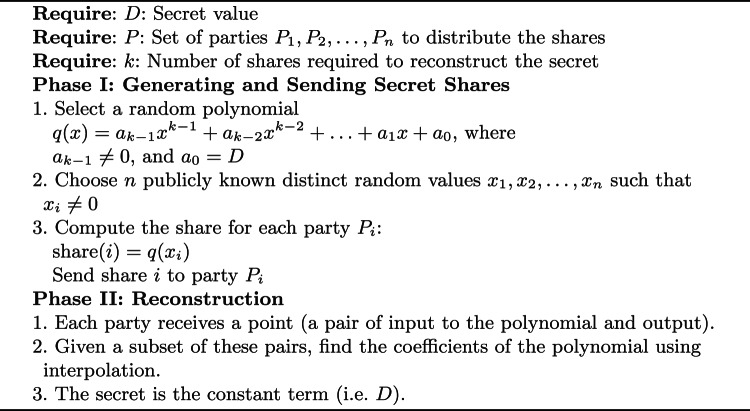
Shamir’s Secret Sharing

**ObliVM** [29] is a secure computation framework that utilises a Java-like language called ObliVM-lang, alongside a two-party garbled circuit protocol. It incorporates a library for both arbitrary-sized and fixed-size integers and features an efficient built-in Oblivious RAM (ORAM) scheme. The framework is designed to perform complex arithmetic operations efficiently, showcasing its capabilities through various illustrative examples.

**GraphSC** [34] is a secure computation framework designed for graph data. It builds upon ObliVM [29], which utilises garbled circuits. The framework allows two parties to compute over shared secret data in parallel, with one party acting as the garbler, responsible for creating the garbled circuits, and the other as the evaluator, who executes these circuits. The graph is represented as tuples, and an oblivious sort is implemented to ensure privacy during computations.

**Sharemind** [11] is a privacy-preserving computation framework that prioritises speed and security. It uses a three-party hybrid protocol with additive secret-sharing over a finite ring, where values are represented as triples. The framework involves clients providing input values, servers performing computations, and outputs receiving the results.

**ABY** [15] is a secure two-party computation framework that enhances efficiency by combining garbled circuits with Yao’s protocol. It uses a hybrid approach involving three protocols: Arithmetic, which uses additive sharing with multiplicative triples on arithmetic circuits; Boolean, which employs an XOR-based sharing scheme; and an optimised version of Yao’s garbled circuit protocol. This combination improves both performance and security over traditional garbled circuit methods [52].

**MP-SPDZ** [26] is a flexible SMPC framework that supports various protocols and optimisations for secure multi-party computations. It includes a preprocessing phase to generate reusable secret shares, reducing computational overhead. The framework also supports multiple communication protocols and integrates key cryptographic primitives like encryption and homomorphic encryption.

**TinyGarble2** [25] is a high-performance framework for privacy-preserving computation using Yao’s Garbled Circuit protocol. It includes components for secure applications and is optimised for speed and memory efficiency, particularly excelling in computations involving Convolutional Neural Networks (CNNs). TinyGarble2 is notably faster and more scalable than other frameworks, with performance up to 18 times better than ObliVM [29].

In this work, we focus on using JIFF [3] as the backend for applying SMPC protocols in our proposed system, PPMQ.

### SMPC for Data Processing

There have been initiatives to utilise SMPC with databases to secure data. For example, a study by Volgushev et al. [48] introduces Conclave, a query compiler specifically designed for relational databases. Conclave works by transforming queries into a series of concise SMPC steps, allowing for local cleartext processing in a data-parallel manner. This approach reduces the reliance on SMPC processing, which is often time-consuming, and improves scalability. The modified queries are then sent to JIFF, which acts as the backend SMPC system [3].

The authors of [8] propose SMCQL, a system that transforms SQL queries into secure multi-party computations. In this system, the user submits their query to a reliable and trustworthy broker. The broker, assumed to be honest, translates the query into a secure computation within a cluster and then sends the result back to the user. In a subsequent research of the SMCQL system [10], researchers used SMCQL as a foundation to develop the SAQE system, which enhances the security of SQL queries. SAQE employs a two-stage process: on the client side, it manages query planning and optimisation, while on the server side, SMPC is utilised to execute queries among data owners. The data owners collaboratively execute the queries, and the server then returns the results to the client. Similarly, Bater et al. expanded on the SMCQL system with the introduction of Shrinkwrap [9]. This approach involves two data owners and utilises two-party secure computations. Although Shrinkwrap improves the performance of SMCQL, it has the limitation of revealing some information during the process.

Moreover, in a publication by Poddar et al. [38] the Senate system is introduced, which enables multiple participants to collaboratively execute analytical SQL queries while keeping their individual data private. A key strength of this system is its capability to defend against malicious parties, a feature that was missing in earlier systems based on a semi-honest architecture.

Furthermore, Secrecy [28] presents a relational SMPC system based on replicated secret sharing. The approach works by splitting the data into three parts: s1, s2, and s3. Each participant holds two of these shares and runs a segment of the query execution code.

Scape [21] is a scalable analytics system for private databases that ensures security against malicious parties. It enables secure database sharing among three non-colluding parties, allowing users to perform various SQL queries, including joins, group by, and aggregations. Benchmark results show that Scape is up to 25 times faster than Secrecy [28].

Waldo [14] is a secure time-series database that uses Function Secret Sharing (FSS) to protect data from untrusted servers while enabling private query execution. It distributes data securely among parties and allows querying through an interface that keeps data hidden. Evaluations with real-world datasets show Waldo’s scalability and superior performance compared to existing systems, highlighting its strong security features for confidential data storage and analysis across various applications.

VaultDB [39] is a framework designed for secure SQL query computation on private data from multiple sources, leveraging the EMP-toolkit for as SMPC backend. The framework was tested on a Clinical Research Network dataset containing nearly 13 million patient records, demonstrating its efficiency and scalability in distributed research while preserving patient privacy without the need for data transfer.

The authors of [50] developed Secure Yannakakis, a secure adaptation of the Yannakakis algorithm specifically designed for computing free-connex join-aggregate queries within a two-party computation model. This protocol preserves data confidentiality during query evaluation, ensuring that individual data remains private. The results demonstrate improved efficiency compared to traditional approaches that rely on Yao’s garbled circuit.

In bioinformatics, the authors of [43] introduce Sequre, a Python based framework for SMPC. It is designed to enhance the performance of SMPC applications by incorporating automatic compile-time optimisations, making computations faster and more efficient. Using secret sharing, Sequre enables the secure processing of biomedical data, improving the speed of queries and analysis tasks.

Hu-Fu [44] is the first system designed for efficient and secure spatial query processing in data federations. It optimises the balance between plaintext and secure operations, reducing the need for secure operators while maintaining overall security. Hu-Fu interprets federated spatial queries in SQL, decomposes them into secure and plaintext parts, and securely combines the results.

In all previous works, the discussion revolves around the use of SMPC within multi-party queries. In contrast, the authors of [22, 51] and [12] investigate the use of SMPC for single-party querying. The SDB system, discussed in [22, 51], is a cloud database system designed for relational tables. It involving two parties: the data owner (DO) and the server provider (SP). Each sensitive data is divided into two parts: an item key held by the DO and ciphertext held by the SP. When a user submits an SQL query, the SDB proxy on the DO side converts queries on sensitive columns into corresponding User-Defined Functions (UDFs) executed at the SP. The modified queries are processed by the SP, which sends back encrypted results to the SDB proxy, where they are decrypted and presented to the user.

Similarly, in [12], the authors present the GOOSE framework, which uses SMPC secret sharing to secure data outsourcing in Resource Description Framework (RDF) databases. In this approach, the data owner uploads graph data to the cloud, dividing it into three distinct parts, each sent to separate locations within an encrypted cloud environment. These components function as part of a multi-party system, ensuring that no single entity has complete knowledge of the entire graph, the query, or the results.

A review of the existing literature reveals that while SMPC has been employed in both relational and graph databases, the concept of multi-party queries specifically for graph databases is relatively new. The initial approach to securing multi-party queries in graph databases was introduced in our previous work through the SMPQ framework [1]. Subsequent developments transformed SMPQ into a fully automated solution, enhancing its capability to handle a broader range of queries with significantly improved performance [2]. This system is notably built upon the Conclave framework, which utilises the SMPC protocol to secure relational databases.

In this work, we present PPMQ [4], a system designed to secure multiparty queries on graph databases. The PPMQ model is an improved solution that addresses the overhead associated with the SMPQ model by removing the Conclave layer, which introduced unnecessary complexity and performance bottlenecks. By eliminating this layer and establishing a direct connection to the JIFF server [3], the system’s performance is significantly enhanced. PPMQ is implemented in JavaScript and built on top of the JIFF library, which provides the implementation of SMPC protocols.

Table 1 provides a comparison between PPMQ and existing systems, highlighting the unique features and advantages of the proposed system.

**Table 1:** SMPC for data processing

Framework	Parties supported	SMPC	Framework backend	Trust Party	No.Data owners	Data model	Query language/API	Available implementation	Development language
Conclave [48]	$>=2$	Secret Sharing	JIFF	Yes	$>=2$	Relational DB	SQL/LINQ	Yes	Python
Congregation	$>=2$	Secret Sharing	JIFF	No	$>=2$	Relational DB	SQL	Yes	Python
SMCQL [8]	2	Garbled Circuits/ ORAM	ObliVM	No	2	Relational DB	SQL	Yes	Java
Senate [38]	2	Garbled Circuits	N/A	No	2	Relational DB	SQL	No	–
SAQE [10]	2	Garbled Circuits	ObliVM	No	2	Relational DB	SQL	No	–
Shrinkwarp [9]	2	Garbled Circuits/ ORAM	ObliVM	No	2	Relational DB	SQL	No	–
Secrecy [28]	3	Repl.Secret Sharing	N/A	No	3	Relational DB	SQL	No	C
Waldo [14]	3	Repl.Secret Sharing	MP-SPDZ	No	3	Time-series DB	MQL	Yes	C++
SecureYannakakis [50]	2	Garbled Circuits	N/A	No	2	Relational DB	SQL	Yes	C++
VaultDB [39]	2	Garbled Circuits	EMP-Toolkit	No	2	Relational DB	SQL	No	C++
Scape [21]	3	Repl.Secret Sharing	Frigate	No	3	Relational DB	SQL	No	–
Sequre [43]	3	Secret Sharing	N/A	Yes	3	Relational DB	SQL	Yes	Python/C++
Hu-Fu [44]	$>=2$	N/A	N/A	Yes	n	Relational DB	SQL	Yes	Java
SMPQ [1]	$>=2$	Secret Sharing	Conclave/JIFF	No	$>=2$	GraphDB	Cypher	No	Python
SDB [22, 51]$^\textrm{a}$	N/A	Secret Sharing	N/A	No	1	Relational DB	SQL	No	–
GOOSE [12] $^\textrm{b}$	N/A	Secret Sharing	N/A	No	1	GraphDB	SPARQL	Yes	Python
**PPMQ** [4]	$>=2$	Secret Sharing	JIFF	No	$>=2$	GraphDB	Cypher	No	JavaScript

*Both $^\textrm{a}$ and $^\textrm{b}$ use SMPC as backend over a database; they do not support multi-party user queries

## PPMQ Overview

The system for efficient privacy-preserving multi-party querying (PPMQ) over federated graph databases offers a customisable and adaptable approach to privacy preservation using two distinct security protocols. The framework is built on top of JIFF [3], a JavaScript library designed for scenarios involving distributed data among multiple entities, enabling SMPC protocols. It employs a server to manage encrypted messages exchanged between participants, operating under an honest-but-curious security model [47]. The system integrates multiple graph databases from different data owners and executes joint queries using the Cypher language, combined with Neo4j Fabric and the APOC library. This section provides an overview of PPMQ, detailing the framework, system requirements, and threat model.

### PPMQ Framework

**Fig. 4:**
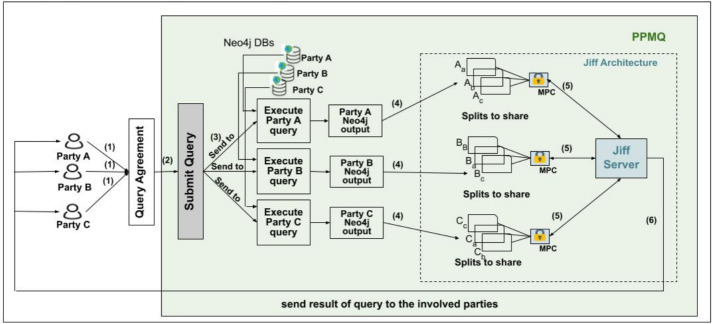
PPMQ architecture and components [4]

Figure 4 presents the entities and their interactions in the framework:

1. **Data owners:** This entity allows multiple data owners, represented as $P_1$, $P_2$,..., $P_n$, to collaboratively execute a joint query across the combined data from their own private databases, after reaching an agreement.
2. **Neo4j Databases:** Each data owner provides access to their respective Neo4j database, allowing the system to execute the sub-query for each party using their individual Neo4j databases.
3. **JIFF Server:** This entity represents a server that offers SMPC protocols, such as secret sharing, to facilitate joint querying [3].

### PPMQ Requirements

The PPMQ framework must meet three key requirements [45]:

- **Privacy:** It involves maintaining the confidentiality of each owner’s private data while facilitating collaborative computing.
- **Efficiency:** It involves how well the system performs, which is affected by the amount of additional communication and computation required.
- **Correctness:** It pertains to conducting accurate analysis in the presence of multiple distributed datasets.

### Threat Model

1. **Data Owners:** Full trust is required among data owners as they collaborate on the query. They must agree to share and process their data securely, trusting that each owner will follow the agreed protocol to ensure data privacy and integrity.
2. **JIFF Server:** This entity also follows a semi-honest security model, which means parties adhere to the protocol but may attempt to learn private information by analysing their messages or colluding with other parties [3]. It ensures that data is split into shares and computations are performed on these shares without revealing the original data.

As in most SMPC-based systems, we assume the semi-honest (honest-but-curious) model where all parties follow the protocol but may try to infer additional information from intermediate results. While this assumption is common in the literature, it does not cover adversarial behaviors such as malicious parties deviating from the protocol, collusion between multiple parties, or active attacks such as result poisoning. Extending PPMQ with protections against malicious adversaries (e.g., using zero-knowledge proofs or verifiable computation) is an important direction for future work.

## How PPMQ Works

This section explains how PPMQ operates, highlighting the two protocols that the system supports: the client-based protocol and the server-based protocol. It begins with the initial step common to both, known as the query agreement, and then illustrates the necessary operations that can be used in both protocols before delving into the details of each. The choice between these protocols depends on the specific computations the parties wish to perform within the query.

### Query Agreement

The PPMQ system enables joint querying by two or more parties. After determining the number of parties involved in performing the query using the SMPC protocol, they must agree on a computation ID (ComID), following the JIFF framework [3]. This ComID serves as an agreement to apply the query across their respective databases.

### Preliminaries

Before explaining the two protocols, it is essential to describe the operations that are used in the protocols descriptions.

The first operation, $Share_n(R)$, is used to split the result R of a query Q into $n$ shares, where $n$ represents the number of parties.

$$ Share_n(R) := (s_1, s_2,\ldots , s_n) $$

If R is a single numerical value then $Share_{n}(R)$ denotes shares obtained by Shamir’a Secret Sharing protocol.

The second operation, $F$, is a function that applies an operation to the shares from different results. This operation can be addition, multiplication, division, comparison, sorting, or intersection.

$$ F(R_{1, s_1}, R_{2, s_2}, \ldots , R_{n, s_n}) $$

For a simple value $x$, we denote by $\text {SHA-256}(x)$ and $\text {HMAC}(x, k)$, the results of applications of hashing algorithms $\text {SHA-256}$ [18] and $\text {HMAC}$ [27] with key $k$ to $x$, respectively . If $x$ is an array:

$$ x = [x_1, x_2, \ldots , x_n] $$

then

$$\begin{aligned} \text {SHA-256}(x):= [\text {SHA-256}(x_1), \text {SHA-256}(x_2), \dots , \text {SHA-256}(x_n)] \end{aligned}$$

and

$$\begin{aligned} \text {HMAC}(x, k):= [\text {HMAC}(x_1, k), \text {HMAC}(x_2, k), \dots , \text {HMAC}(x_n, k)]. \end{aligned}$$

In the protocol, to enhance security and conceal the results of the hash values, we used the a key ($DH$) exchangesd by Diffie-Hellman key exchange [30] as follows:

$$\begin{aligned} & \text {SHA-256}(x)\oplus DH:= [\text {SHA-256}(x_1) \oplus DH,\\ & \quad \text {SHA-256}(x_2) \oplus DH,\ldots , \text {SHA-256}(x_n) \oplus DH] \end{aligned}$$

and

$$\begin{aligned} & \text {HMAC}(x, DH):= [\text {HMAC}(x_1, DH), \\ & \quad \text {HMAC}(x_2, DH), \dots , \text {HMAC}(x_n, DH)] \end{aligned}$$

### Supported Query Operations and Scope

The PPMQ framework supports federated queries through two distinct protocols, each catering to different types of operations. In its current form, PPMQ supports the class of *conjunctive queries (CQs)*, which correspond to Select–Project-Join (SPJ) queries without negation or disjunction, optionally extended with simple aggregation operations such as COUNT and SUM. In other words, the queries supported are those that can be decomposed into sub-queries executable independently on each participating database, followed by a single secure computation phase on the combined results. This design naturally aligns with Basic Graph Patterns (BGPs) in Cypher, where all MATCH clauses can be flattened into one round of joins.

These protocols facilitate various secure computations. Specifically, the client-based protocol enables arithmetic, comparative, and set-theoretic operations, while the server-based protocol provides efficient private set intersection. The current system, therefore, focuses on these foundational single-round query capabilities, covering the practical SPJ(+A) fragment of graph workloads. Consequently, more advanced Cypher query features, such as traversal queries that generally require multiple rounds of execution, were not tested in this version as they were beyond the scope of this study. However, in our ongoing work, we are extending the system to support unions of conjunctive queries (UCQs) and property path queries (such as reachability or transitive closure), enabling more expressive workloads under the same privacy-preserving framework. Preliminary results have appeared in [5].

### Protocol 1: Client-Based

The first protocol in the PPMQ system operates on the client side, utilising traditional SMPC protocols. This protocol enables computations to be performed on encrypted data, ensuring that sensitive information remains confidential. The class of queries supported by the protocol includes all Cypher queries which are instances of the following template.
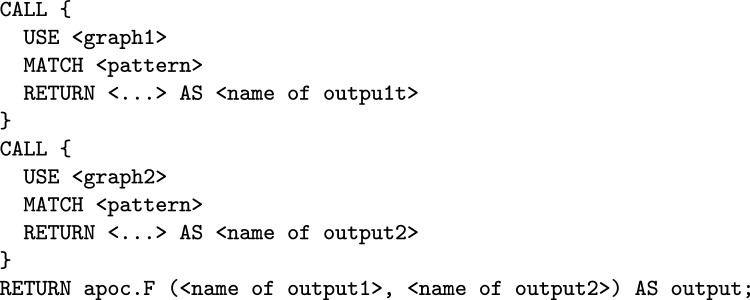


Where $F$ can represent operations such as addition, multiplication, division, comparison, intersection or any other operation supported by the APOC library.

In this protocol, traditional SMPC methods, specifically Secret Sharing, are applied on the client side by leveraging the functionality provided by JIFF. The sub-queries results from each party are transmitted to the JIFF server, where they are split into shares. The server then stores and routes encrypted messages exchanged among the participating parties, ensuring that each party receives one share from the other. Each party computes an intermediate result using the share it received. In the final reconstruction phase, the parties exchange their intermediate results and combine them to determine the final result of the joint query. This process allows them to collaboratively compute the joint function without disclosing their inputs.

JIFF’s functionality includes performing arithmetic operations like secure addition, multiplication, and division of secret-shared numbers. It also supports comparison and sorting operations, allowing parties to establish the relative magnitude and order of their inputs without disclosing the actual values. Additionally, a custom function has been developed to identify the intersection between two or more secret-shared strings, further extending JIFF’s functionality. Algorithm 2 illustrates the phases involved in the client-based query protocol.

**Algorithm 2:**
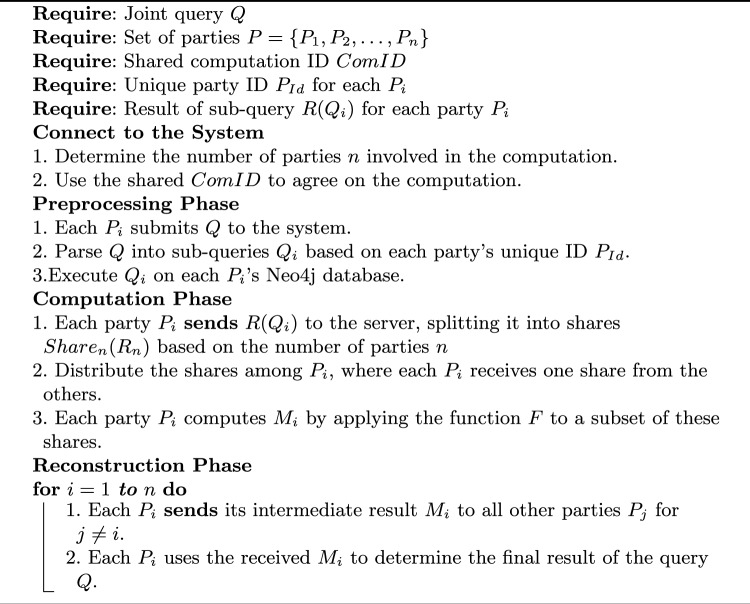
PPMQ Client-Based Query Execution for $n$ Parties

The following notation illustrates the steps of the client-based protocol, showing the communication steps using the notation detailed in subsection 5.2:

$$ \begin{array}{l} \text {Party 1:} \quad R_1 {{:}{=}}\textrm{r}(Q_1); \; (R_{1,1}, R_{1,2}, R_{1,3}) {{:}{=}}\textrm{Share}_3(R_1); \; \textbf{Send}(S, [R_{1,1}, R_{1,2}]) \\ \text {Party 2:} \quad R_2 {{:}{=}}\textrm{r}(Q_2); \; (R_{2,1}, R_{2,2}, R_{2,3}) {{:}{=}}\textrm{Share}_3(R_2); \; \textbf{Send}(S, [R_{2,1}, R_{2,2}]) \\ \text {Party 3:} \quad R_3 {{:}{=}}\textrm{r}(Q_3); \; (R_{3,1}, R_{3,2}, R_{3,3}) {{:}{=}}\textrm{Share}_3(R_3); \; \textbf{Send}(S, [R_{3,1}, R_{3,2}]) \\ \text {Server } S: \quad \textbf{Send}\big ((P_1, [R_{2,1}, R_{3,1}]); \; (P_2, [R_{1,1}, R_{3,2}]); \; (P_3, [R_{2,2}, R_{1,2}])\big ) \\ \text {Party 1:} \quad M_1 {{:}{=}}\textrm{F}(R_{1,3}, R_{2,1}, R_{3,1}); \quad \textbf{Send}(M_1, [P_2, P_3]) \\ \text {Party 2:} \quad M_2 {{:}{=}}\textrm{F}(R_{2,3}, R_{1,1}, R_{3,1}); \quad \textbf{Send}(M_2, [P_1, P_3]) \\ \text {Party 3:} \quad M_3 {{:}{=}}\textrm{F}(R_{3,3}, R_{1,1}, R_{2,1}); \quad \textbf{Send}(M_3, [P_1, P_2]) \\ \text {Each Party } P_1, P_2, P_3: \quad C {{:}{=}}\textrm{F}(M_1, M_2, M_3); \end{array} $$

### Protocol 2: Server-Based

In an implementation of set intersection protocol on a client side each party receives shares from the other parties (without knowing the source) and identifies duplicate shares as the intersection. While it provides some degree of anonymity protection, it also leaks the private information to other parties. To address this issue, an alternative protocol for private set intersection is proposed, which is implemented on the server side. Thus, the class of queries supported by the protocol includes all Cypher queries which are instances of the following template.
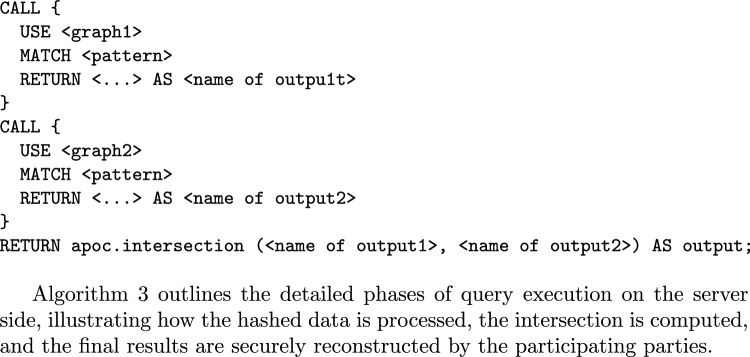


Algorithm 3 outlines the detailed phases of query execution on the server side, illustrating how the hashed data is processed, the intersection is computed, and the final results are securely reconstructed by the participating parties.

**Algorithm 3:**
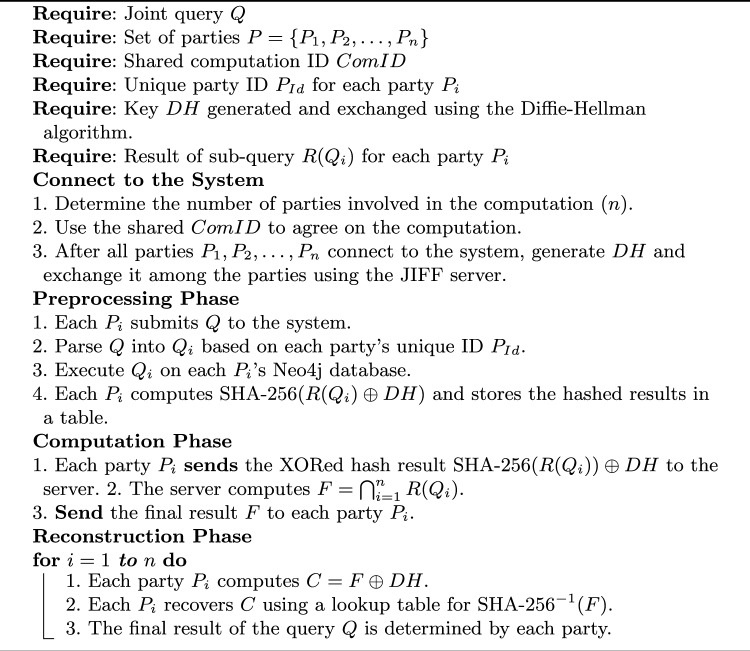
PPMQ Server-Based Query Execution

In this protocol, data is first hashed using SHA-256. This hashing process generates a fixed-size 256-bit hash value from the input data. These hashed values are then used for computations. This approach ensures that even though the server processes the data, it cannot access the original values, assuming the hashing is not reversible. To further enhance security, a DH key exchange is performed, allowing the parties to securely share a key that is then used to XOR the hashed values. This additional step of XORing with the DH key ensures that even the hashed values are protected against potential server access. This method is particularly effective in scenarios where multiple parties need to find intersections among their private datasets without revealing the actual data to the server.

In addition to the previously mentioned method, an alternative approach can be used for enhanced security involves using HMAC, which combines a hash function (such as SHA-256) with the secret key generated by DH protocol. The server’s ability to perform intersection operations on protected values relies crucially on HMAC being a deterministic function. The changes to the previous protocol Algorithm 3 be only in the preprocessing and reconstruction phases as follows:
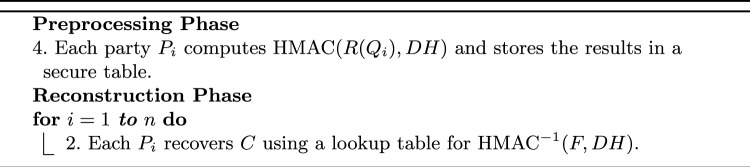


As an enhancement to the system’s functionality, we have modified this protocol to enable secure traversal queries across different parties, as discussed in our paper [5].

The following notation illustrates the steps of the server-based protocol, showing the communication steps:

$$ \begin{array}{l} \text {Party 1:} \quad R_1 {{:}{=}}\textbf{r}(Q_1); \; M_1 {{:}{=}}\text {HMAC}(R_1, \text {DH}); \; \textbf{Send}(S, M_1) \\ \text {Party 2:} \quad R_2 {{:}{=}}\textbf{r}(Q_2); \; M_2 {{:}{=}}\text {HMAC}(R_2, \text {DH}); \; \textbf{Send}(S, M_2) \\ \text {Server:} \quad C {{:}{=}}\textbf{F}(M_1, M_2); \; \textbf{Send}([P_1, P_2], C) \\ \text {Party 1:} \quad F_1 {{:}{=}}\text {HMAC}^{-1}(C, \text {DH}) \\ \text {Party 2:} \quad F_2 {{:}{=}}\text {HMAC}^{-1}(C, \text {DH}) \\ \end{array} $$

## Query Workflow

Figure 4 outlines the query flow within the PPMQ system. Multiple data owners ($P_1$ to $P_n$) collaborate to execute a joint query on their separate graph databases. In step (1), they agree upon a joint query and establish a shared computation ID. In step (2), the parties submit the query, which is then divided into sub-queries according to their respective party IDs. In step (3), each party runs its sub-query on its own Neo4j database. These sub-queries may either be identical across all parties or vary depending on the context. Steps (1) to (3) are executed in a similar manner on both the client and server sides.

### Query Workflow—Client Side

In step (4), the sub-query results are transmitted to the JIFF server, making use of SMPC protocols. Privacy is preserved by passing the results as shares, employing Shamir’s secret sharing technique [42]. In step (5), these shares are distributed among the parties, enabling them to carry out a secure computation and compute the final query results. Finally, in step (6), the final result is disclosed to the parties who initiated the query, ensuring the confidentiality of the underlying data remains intact.

To demonstrate how this operates, imagine a scenario involving three parties attempting to execute one of the queries from subsection 7.1, specifically Q2. This query seeks to **Count the number of students with scores of 7 that are present in all of the databases**. The syntax for the joint query would be structured as follows:
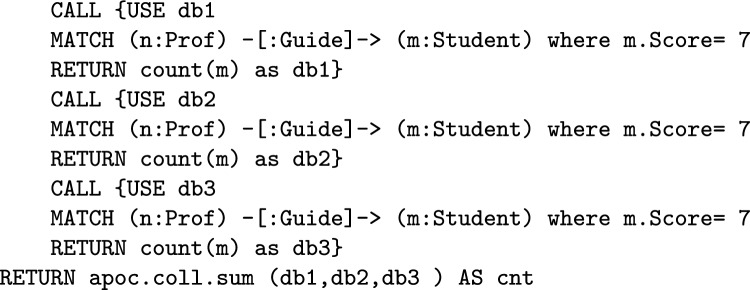


In this example, each party executes a sub-query to count students with a score of 7 in its respective database. The results ($db_1$, $db_2$, $db_3$) are transmitted to the server as shares. These shares are then distributed among the parties to compute intermediate results. Finally, based on these intermediate results, the final result of the joint query $Q$ is determined.

### Query Workflow- Server Side

In Step (4), a random key (Dh) is generated using the Diffe-Hellman key exchange between the parties [30]. In Step (5), once the query results are obtained and before they are passed to the JIFF server, each party hashes its result using HMAC and Dh as secret key. Moving to Step (6), the JIFF server performs SMPC to divide each party’s private data into smaller shares but does not distribute these shares among the parties. Instead, the server carries out the computation to find the intersection. Finally, in Step (7), the final result is revealed to the parties who initiated the query.

To illustrate how this process works, consider the following example involving two parties. They want to execute Query Q7 from 7.2 to find **the names of all the actors in the databases who were born in 1974**. The syntax for the joint query is as follows:
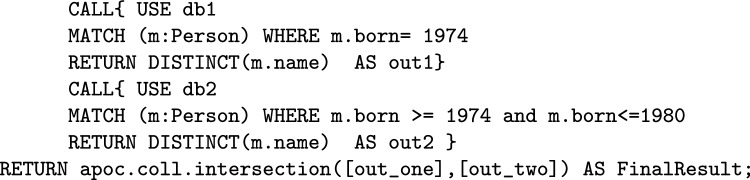


In this example, each party executes a sub-query to identify distinct names based on certain conditions. The results (out1, out2) are sent as shares to a server, where they are hashed using HMAC for added security. The server computes the intersection of these results without revealing the underlying data. The final result of the joint query *Q*, representing the shared names between the parties, is then returned to the participating entities, ensuring privacy-preserving data collaboration.

## System Evaluation

We evaluate the efficiency of our proposed system, PPMQ, addressing the following questions:

- **RQ1**: How effective is the system in ensuring secure multiparty queries, and what is its efficiency in terms of performance?
- **RQ2**: Does the data size impact the system’s efficiency?
- **RQ3**: How does PPMQ’s performance compare to existing systems like Neo4j Fabric, Conclave, and SMPQ?
- **RQ4**: How does the performance of PPMQ scale with an increasing number of parties?

### Data Sets

To evaluate the proposed PPMQ system and measure its efficiency, a total of 15 queries were executed using four distinct datasets of varying sizes to analyse the impact of increasing data volume on performance. These datasets were sourced from three different parties, each utilising separate Neo4j databases.

The first dataset, specifically created for this study, consists of data about professors and students, comprising 58 nodes and 29 edges. This dataset serves as a small-scale example for initial testing. The second dataset is adapted from an open Neo4j example, known as the ’Movie’ dataset [36]. Modifications were made by adding and removing nodes to vary party sizes. This graph contains 563 nodes and 785 edges, enabling a more comprehensive evaluation.

The third dataset, referred to as the ’POLE’ dataset, is a large-scale dataset containing open crime data from Manchester, UK, dated August 2017 [24]. It includes 61,521 nodes and 105,840 relationships. Lastly, the ’Car Location’ dataset was developed using solely numerical data. Across all databases within this dataset, there are 100 nodes connected by 63 relationships. This dataset was specifically created to compare the proposed system with the Conclave system, which is tailored for handling numerical data.

For clarity, the first dataset was utilised for queries Q1 to Q4, while the Movie dataset supported queries Q5 to Q8. Queries Q9 to Q12 were executed using the Car Location dataset, and queries Q13 to Q15 were conducted with the POLE dataset. Table 2 provides further information on each dataset, including the number of nodes and relationships in each database managed by the distinct data owners.

**Table 2:** Details of the four datasets used in the experiments [4]

Dataset	Database	No. of nodes	No. of relationships
Prof-student	DB1	32	16
	DB2	10	5
	DB3	16	8
Movie	DB1	203	269
	DB2	172	254
	DB3	188	262
POLE	DB1	61,521	105,840
	DB2	61,521	105,840
	DB3	61,521	105,840
Car-Location	DB1	44	31
	DB2	24	15
	DB3	32	17

### Queries

Below is the list of the 15 queries used to validate our system.^1^

- **Q1:** Count how many students there are in common between all DBs.
- **Q2**: Count the number of students who scored 7 and are common across all databases.
- **Q3**: Find the names of the common students across all the databases with scores of 9 or above.
- **Q4**: Find names of the common students across all databases with scores of 7.
- **Q5**: Count the number of movies with the actor Tom Hanks that are common across all databases.
- **Q6**: Find the names of the movies that are common across all databases with the actor Tom Hanks.
- **Q7**: Find the names of all actors who were born in 1974.
- **Q8**: Finds the sum of all nodes in the movie DB for all parties.
- **Q9**: Find the number of cars present at each specific location.
- **Q10**: Find the total count of cars with ID=1 across all the databases.
- **Q11**: Combine two databases by utilising the node id of the professors whose students have a grade equal to or higher than 9.0 in either of the databases.
- **Q12**: Retrieve information from two databases by selecting the scores of all students enrolled in the Math course from both databases.
- **Q13**: Find the total number of robbery crimes across all databases for the year 2017 in Manchester.
- **Q14**: Determine the location with the highest frequency of recorded burglaries in common across all databases.
- **Q15**: Find which crime Inspector Morse investigated is listed in all databases.

### Experiments Result

**Fig. 5:**
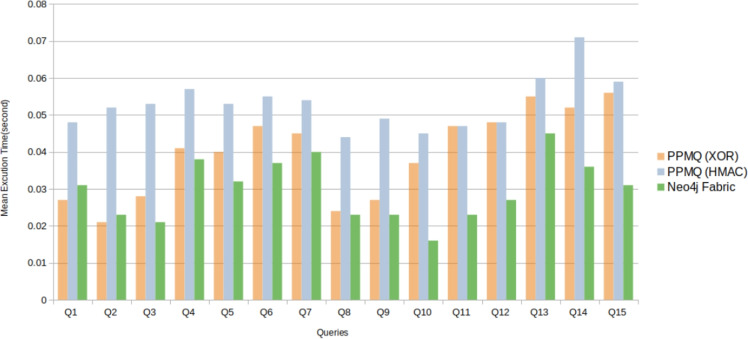
Execution times for Q1–Q15 when using PPMQ and Neo4j

The experiments were conducted on a desktop PC running Windows 11 with an Intel Core i7 processor clocked at 1.5 GHz and 16.00 GB of RAM, with three parties utilising a local database for each party. Execution times, representing the duration for all parties to obtain query results, were measured using the *performance.now()* function in JavaScript, providing high-resolution timestamps in milliseconds. For each query, 10 iterations were executed, and the mean execution times, the standard deviations (SD), and the relative standard deviations (RSD) for all queries were calculated. Table 3 and Figure 5 showcase the mean running times, SD, and RSD for all queries, comparing PPMQ with Neo4j Fabric, the popular framework for federated graph databases which does not use any SMPC protocols.

**Table 3:** Execution times for queries using PPMQ (XOR Hash), PPMQ (HMAC), and Neo4j fabric

Query	PPMQ (XOR)			PPMQ (HMAC)			Neo4j Fabric		
	Mean	SD	RSD	Mean	SD	RSD	Mean	SD	RSD
**Q1**	0.027	0.002	8.47%	0.048	0.008	16.73%	0.031	0.003	12.62%
**Q2**	0.021	0.002	11.98%	0.052	0.008	16.07%	0.023	0.001	7.22%
**Q3**	0.028	0.003	11.10%	0.053	0.006	12.83%	0.021	0.005	24.72%
**Q4**	0.041	0.008	21.01%	0.057	0.008	14.15%	0.038	0.003	9.03%
**Q5**	0.040	0.005	13.38%	0.053	0.010	18.77%	0.032	0.003	11.02%
**Q6**	0.047	0.006	12.95%	0.055	0.007	12.40%	0.037	0.002	6.62%
**Q7**	0.045	0.007	17.01%	0.054	0.012	22.76%	0.040	0.007	19.54%
**Q8**	0.024	0.002	8.21%	0.044	0.005	11.77%	0.023	0.005	22.36%
**Q9**	0.027	0.003	12.23%	0.049	0.007	14.58%	0.023	0.005	23.34%
**Q10**	0.037	0.004	11.19%	0.045	0.005	12.42%	0.016	0.001	11.93%
**Q11**	0.047	0.005	11.60%	0.047	0.007	15.74%	0.023	0.006	26.13%
**Q12**	0.048	0.006	12.94%	0.048	0.008	15.80%	0.027	0.005	20.07%
**Q13**	0.055	0.004	8.10%	0.060	0.007	12.31%	0.045	0.006	14.41%
**Q14**	0.052	0.004	9.38%	0.071	0.009	12.16%	0.036	0.004	11.82%
**Q15**	0.056	0.006	11.14%	0.059	0.006	11.68%	0.031	0.004	14.82%

The results reveal that both approaches of PPMQ (Hash XOR and HMAC)and Neo4j Fabric demonstrate comparable execution times, with PPMQ generally taking slightly longer processing durations. Notably, even when executing queries Q13 to Q15 on a large dataset, PPMQ’s performance remains within an acceptable range of 52 to 56 ms for the first approach (Hash XOR) and 59 to 71 ms for the second approach (HMAC). In comparison, Neo4j Fabric executes the same queries more faster, with execution times ranging from 31 to 45 ms. When contrasting the two PPMQ approaches, the Hash XOR method provides marginally faster execution times compared to HMAC. However, HMAC offers enhanced security features, which may justify the longer execution time. The evaluation covered a range of queries to assess the performance of PPMQ across different query types, addressing RQ1. Furthermore, the impact of data size on system efficiency was explored using the large-scale ’POLE’ dataset, addressing RQ2.

**Fig. 6:**
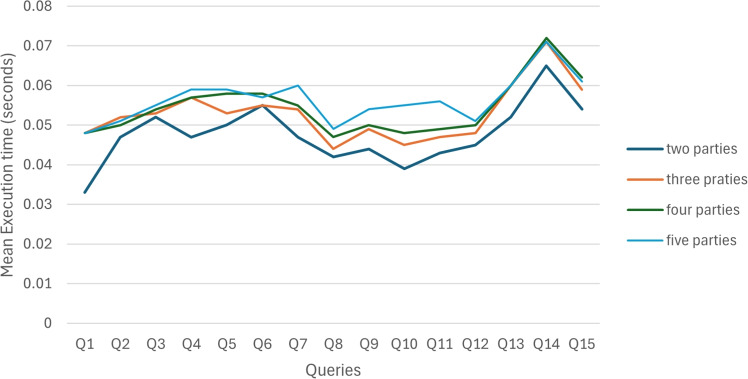
Scalability of PPMQ with varying party count

To evaluate the performance of PPMQ and how it scales with an increasing number of parties, we executed the same queries mentioned in 7.2 under different settings: two, three, four, and five parties. The table labeled 4, along with the accompanying Fig. 6, provides a comparative analysis of the PPMQ system’s performance, highlighting the mean execution times (in milliseconds) for various queries (Q1 to Q15) as the number of participating parties increases. The table shows a comparative analysis of the performance of the PPMQ system as the number of parties increases from two to five. Overall, the system’s execution times for queries Q1 to Q15 generally increase with the number of participating parties. This indicates that the PPMQ system experiences a scaling effect where more parties result in slightly longer execution times, though the increase is gradual and remains within an expected range.

**Table 4:** Execution times for Q1–Q15 using the PPMQ system with varying numbers of parties

PPMQ system				
	Two parties	Three parties	Four parties	Five parties
Query	Mean (s)			
**Q1**	0.033	0.048	0.048	0.048
**Q2**	0.047	0.052	0.050	0.051
**Q3**	0.052	0.053	0.054	0.055
**Q4**	0.047	0.057	0.057	0.059
**Q5**	0.050	0.053	0.058	0.059
**Q6**	0.055	0.055	0.058	0.057
**Q7**	0.047	0.054	0.055	0.060
**Q8**	0.042	0.044	0.047	0.049
**Q9**	0.044	0.049	0.050	0.054
**Q10**	0.039	0.045	0.048	0.055
**Q11**	0.043	0.047	0.049	0.056
**Q12**	0.045	0.048	0.050	0.051
**Q13**	0.052	0.060	0.060	0.060
**Q14**	0.065	0.071	0.072	0.071
**Q15**	0.054	0.059	0.062	0.061

### Comparison with Prior Work

This subsection aims to compare the PPMQ system with two existing systems that utilise SMPC for data processing. The first system, Conclave [48], employs SMPC to secure relational databases and serves as the backend for the second system, SMPQ [1], which focuses on secure multiparty queries in graph databases. To ensure a fair comparison with Conclave, the same queries from the experiments were executed using their SQL equivalents.

As mentioned in previous published papers [1] and [2], Conclave only supports numerical data. Consequently, queries Q1-Q8 and Q13-Q15 could not be executed. To address this limitation, the "Car location" dataset, which exclusively consists of numerical data, was used to perform queries Q9-Q11.

**Fig. 7:**
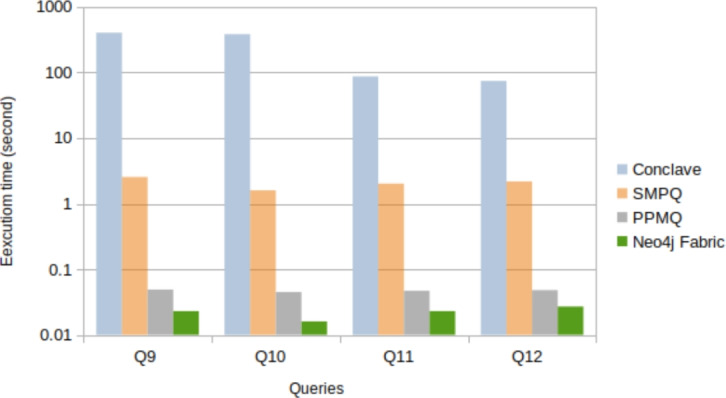
Execution times for Q9–Q12 when using conclave, SMPQ, PPMQ, and Neo4j

Running these queries in the Conclave system resulted in execution times ranging from approximately 402 to 74 s. Subsequently, executing the same queries in the SMPQ system reduced the time to 1.6 to 2.5 s. This performance improvement was achieved by eliminating the sorting function used in the Conclave system after obtaining the query results and executing them simultaneously for all parties. In comparison, PPMQ demonstrated a significant advancement in query execution time, taking nearly less than a second, which is comparable with Neo4j Fabric. Table 5 and Figure 7 illustrate the comparison of execution times when running the mentioned queries using Conclave, SMPQ, PPMQ, and Neo4j Fabric.

**Table 5:** Execution times of Q9–Q12: conclave, SMPQ,PPMQ, and Neo4j fabric

Query	Conclave(s)	SMPQ(s)	PPMQ(s)	Neo4j fabric(s)
**Q9**	402.3	2.55	0.049	0.023
**Q10**	382.3	1.60	0.045	0.016
**Q11**	86.88	2.02	0.047	0.023
**Q12**	74.1	2.18	0.048	0.027

## Conclusion

We have developed a framework to secure multi-party queries over federated graph databases, utilising SMPC protocols. The system is built with the JIFF server as the backend for implementing these protocols. Additionally, we have extended the system’s capabilities to automatically parse queries based on the party ID, ensuring that each party executes the appropriate sub-query.

In future work, we aim to adapt the system for querying heterogeneous federated databases, where multiple parties contribute their private databases for joint querying, all while maintaining privacy and security through SMPC protocols.

## Data Availability

The datasets generated and analysed during the current study are available from the corresponding author upon request via email.

## Footnotes

1 all Cypher queries can be found in the Appendix available at https://doi.org/10.5281/zenodo.11048030

## References

[1] Al-Juaid N, Lisitsa A, Schewe S. SMPG: Secure multi party computation on graph databases. In: ICISSP; 2022. pp. 463–471.

[2] Al-Juaid N, Lisitsa A, Schewe S. Secure joint querying over federated graph databases utilising SMPC protocols. In: Proceedings of the 9th International Conference on Information Systems Security and Privacy - Volume 1: ICISSP; 2023. pp. 210–217. INSTICC, SciTePress.

[3] Albab KD, Issa R, Lapets A, Flockhart P, Qin L, Globus-Harris I. . Tutorial: Deploying secure multi-party computation on the web using JIFF. In: 2019 IEEE Cybersecurity Development (SecDev); 2019. pp 3–3. IEEE.

[4] Aljuaid, N, Lisitsa, A, Schewe S. Efficient and secure multiparty querying over federated graph databases. In: Proceedings of the 13th International Conference on Data Science, Technology and Applications—DATA; 2024a. pp. 39–50. INSTICC, SciTePress.

[5] Aljuaid N, Lisitsa A, Schewe S. Secure multi-party traversal queries over federated graph databases. In: Proceedings of the 21st International Conference on Security and Cryptography - SECRYPT; 2024b. pp. 716–721. INSTICC, SciTePress.

[6] Alwen J, Ostrovsky R, Zhou H, Zikas V. Incoercible multi-party computation and universally composable receipt-free voting. In: Advances in Cryptology-CRYPTO 2015: 35th Annual Cryptology Conference, vol. 9216. Berlin Heidelberg: Springer; 2015. pp. 763–80.

[7] Aly A, Van Vyve M. Practically efficient secure single-commodity multi-market auctions. In: International Conference on Financial Cryptography and Data Security. Berlin: Springer; 2016. pp. 110–129.

[8] Bater J, Elliott G, Eggen C, Goel S, Kho A, Rogers J. SMCQL: secure querying for federated databases 2016. arXiv:1606.06808.

[9] Bater J, He X, Ehrich W, Machanavajjhala A, Rogers J. Shrinkwrap: efficient SQL query processing in differentially private data federations. Proc VLDB Endow. 2018;12(3):307–20.

[10] Bater J, Park Y, He X, Wang X, Rogers J. SAQE: practical privacy-preserving approximate query processing for data federations. Proc VLDB Endow. 2020;13(12):2691–705.

[11] Bogdanov D, Laur S, Willemson J Sharemind: A framework for fast privacy-preserving computations. In: Computer Security-ESORICS 2008: 13th European Symposium on Research in Computer Security, Málaga, Spain, 6–8 Oct 2008; 2008. pp. 192–206.

[12] Ciucanu R, Lafourcade P. GOOSE: A secure framework for graph outsourcing and sparql evaluation. In: 34th Annual IFIP WG 11.3 Conference on Data and Applications Security and Privacy (DBSec’20). Accepté, à paraître 2020.

[13] Cramer R, Damgård IB, Nielsen JB. Secure multiparty computation. Cambridge University Press. 2015.

[14] Dauterman E, Rathee M, Popa RA, Stoica I. Waldo: A private time-series database from function secret sharing. Cryptology ePrint Archive, Paper 2021/1661 2021. https://eprint.iacr.org/2021/1661.

[15] Demmler D, Schneider T, Zohner M. Aby-a framework for efficient mixed-protocol secure two-party computation. In: NDSS 2015.

[16] Evans D, Kolesnikov V, Rosulek M. A pragmatic introduction to secure multi-party computation. Found Trends Priv Secur. 2018;2:70–246.

[17] Francis N, Green A, Guagliardo P, Libkin L, Lindaaker T, Marsault V, Plantikow S, Rydberg M, Selmer P, Taylor A. Cypher: An evolving query language for property graphs. In: Proceedings of the 2018 International Conference on Management of Data; 2018. pp. 1433–1445.

[18] Gilbert H, Handschuh H. . Security analysis of sha-256 and sisters. In: International workshop on selected areas in cryptography; 2003. pp. 175–193.

[19] Gu Z, Corcoglioniti F, Lanti D, Mosca A, Xiao G, Xiong J, Calvanese D. (to appear). A systematic overview of data federation systems. Semantic Web.

[20] Guia J, Soares VG, Bernardino J. Graph databases: Neo4j analysis. In: ICEIS. 2017;1:351–356.

[21] Han F, Zhang L, Feng H, Liu W, Li X. Scape: Scalable collaborative analytics system on private database with malicious security. In: 2022 IEEE 38th International Conference on Data Engineering (ICDE); 2022, 1740–1753. IEEE.

[22] He Z, Wong WK, Kao B, Cheung D, Li R, Yiu S, et al. SDB: A secure query processing system with data interoperability. Proc VLDB Endow. 2015;8:1876–9.

[23] Hemenway B, Welser W IV, Baiocchi D. Achieving higher-fidelity conjunction analyses using cryptography to improve information sharing. Technical report, Rand project, Air Force: Santa Monica, CA; 2014.

[24] Hunger M. neo4j-graph-examples/pole 2020. https://github.com/neo4j-graph-examples/pole/. Accessed: 12 Dec 2023.

[25] Hussain S, Li B, Koushanfar F, Cammarota R. Tinygarble2: Smart, efficient, and scalable yao’s garble circuit. In: Proceedings of the 2020 Workshop on Privacy-Preserving Machine Learning in Practice; 2020. pp. 65–67.

[26] Keller M. Mp-spdz: A versatile framework for multi-party computation. In: Proceedings of the 2020 ACM SIGSAC conference on computer and communications security; 2020. pp. 1575–1590.

[27] Krawczyk H, Bellare M, Canetti R. Hmac: Keyed-hashing for message authentication. Technical Report RFC 2104, IETF 1997.

[28] Liagouris J, Kalavri V, Faisal M, Varia M. Secrecy: Secure collaborative analytics on secret-shared data 2021. arXiv:2102.01048.

[29] Liu C, Wang XS, Nayak K, Huang Y, Shi E. ObliVM: A programming framework for secure computation. In: 2015 IEEE Symposium on Security and Privacy; 2015. pp. 359–376.

[30] Maurer U, Wolf S. Diffie-hellman, decision diffie-hellman, and discrete logarithms. In: Proceedings. 1998 IEEE International Symposium on Information Theory (Cat. No. 98CH36252); 1998. pp. 327. IEEE.

[31] Miller JJ. Graph database applications and concepts with Neo4j. In: Proceedings of the Southern Association for Information Systems Conference, Atlanta, GA, USA, volume 2324; 2013.

[32] Mood B, Gupta D, Carter H, Butler K, Traynor P. Frigate: A validated, extensible, and efficient compiler and interpreter for secure computation. In: 2016 IEEE European Symposium on Security and Privacy (EuroS &P); 2016. pp. 112–127. IEEE.

[33] Mostafa A. Security of database management systems; 2016. pp. 1–6. https://www.researchgate.net/publication/301613094.

[34] Nayak K, Wang XS, Ioannidis S, Weinsberg U, Taft N, Shi E. Graphsc: Parallel secure computation made easy. In: 2015 IEEE Symposium on Security and Privacy; 2015. pp. 377–394. IEEE.

[35] Needham M, Hodler AE. Graph algorithms: practical examples in Apache Spark and Neo4j. O’Reilly Media 2019.

[36] Neo4j. built-in examples: Movie-graph 2007. https://neo4j.com/developer/example-data/#built-in-examples/. Accessed on 28 Dec 2023.

[37] Neo4j Inc. Neo4j fabric: Operations manual 4.0 2011. https://neo4j.com/docs/operations-manual/4.0/fabric/. Accessed: 05-09-2024.

[38] Poddar R, Kalra S, Yanai A, Deng R, Popa RA, Hellerstein JM. . Senate: A maliciously-secure mpc platform for collaborative analytics. arXiv e-prints; 2020. pp. arXiv–2010.

[39] Rogers J, Adetoro E, Bater J, Canter T, Fu D, Hamilton A, Hassan A, Martinez A, Michalski E, Mitrovic V, et al. Vaultdb: A real-world pilot of secure multi-party computation within a clinical research network 2022; 2022.arXiv:2203.00146.

[40] Salehnia A. Comparisons of relational databases with big data: a teaching approach. Brookings: South Dakota State University; 2017.

[41] Sangers A, van Heesch M, Attema T, Veugen T, Wiggerman M, Veldsink J, Bloemen O, Worm D. Secure multiparty pagerank algorithm for collaborative fraud detection. In: Financial Cryptography and Data Security: 23rd International Conference, FC 2019, Frigate Bay, St. Kitts and Nevis, 18–22 Feb 2019, Revised Selected Papers 23; 2019. pp. 605–623. Springer.

[42] Shamir A. How to share a secret. Commun ACM. 1979;22(11):612–3.

[43] Smajlović H, Shajii A, Berger B, Cho H, Numanagić I. Sequre: a high-performance framework for secure multiparty computation enables biomedical data sharing. Genome Biol. 2023;24(1):1–18. 36631897 10.1186/s13059-022-02841-5 PMC9832703

[44] Tong Y, Pan X, Zeng Y, Shi Y, Xue C, Zhou Z, et al. Hu-fu: Efficient and secure spatial queries over data federation. Proc VLDB Endow. 2022;15(6):1159.

[45] Tong Y, Zeng Y, Zhou Z, Liu B, Shi Y, Li S, Xu K, Lv W. Federated computing: Query, learning, and beyond 2023.

[46] van Egmond MB, Spini G, van der Galien O, A IJpma, Veugen T, Kraaij W, et al. Privacy-preserving dataset combination and lasso regression for healthcare predictions. BMC Med Inform Decis Mak. 2021;21(1):1–16. 10.1186/s12911-021-01582-y PMC8445286 34530824

[47] Veeningen M, Weger B, Zannone N. Modeling identity-related properties and their privacy strength. In: Formal Aspects of Security and Trust, volume 6561 of Lecture Notes in Computer Science; 2011. 126–140. Springer.

[48] Volgushev N, Schwarzkopf M, Getchell B, Varia M, Lapets A, Bestavros A. Conclave: secure multi-party computation on big data. In: Proceedings of the Fourteenth EuroSys Conference. 2019;2019:1–18.

[49] Wang X, Malozemoff AJ, Katz J. Emp-toolkit: Efficient multiparty computation toolkit 2016.

[50] Wang Y, Yi K. Secure yannakakis: Join-aggregate queries over private data. In: Proceedings of the 2021 International Conference on Management of Data, SIGMOD ’21; 2021. pp. 1969–1981. New York, NY, USA. Association for Computing Machinery.

[51] Wong WK, Kao B, Cheung D. WL, Li R, Yiu SM. Secure query processing with data interoperability in a cloud database environment. In: Proceedings of the 2014 ACM SIGMOD international conference on Management of data; 2014. p. 1395–1406.

[52] Yao AC. Protocols for secure computations. In: Proceedings of the 23rd Annual ACM Symposium on Theory of Computing (STOC); 1982. pp. 160–164.

